# miRNAmotif—A Tool for the Prediction of Pre-miRNA–Protein Interactions

**DOI:** 10.3390/ijms19124075

**Published:** 2018-12-17

**Authors:** Martyna O. Urbanek-Trzeciak, Edyta Jaworska, Wlodzimierz J. Krzyzosiak

**Affiliations:** Institute of Bioorganic Chemistry, Polish Academy of Sciences, Noskowskiego 12/14, 61-704 Poznan, Poland; e.jaworska@qmul.ac.uk (E.J.); wlodkrzy@ibch.poznan.pl (W.J.K.)

**Keywords:** microRNA, RNA–protein interactions, translation regulation, post-transcriptional regulation, motifs

## Abstract

MicroRNAs (miRNAs) are short, non-coding post-transcriptional gene regulators. In mammalian cells, mature miRNAs are produced from primary precursors (pri-miRNAs) using canonical protein machinery, which includes Drosha/DGCR8 and Dicer, or the non-canonical mirtron pathway. In plant cells, mature miRNAs are excised from pri-miRNAs by the DICER-LIKE1 (DCL1) protein complex. The involvement of multiple regulatory proteins that bind directly to distinct miRNA precursors in a sequence- or structure-dependent manner adds to the complexity of the miRNA maturation process. Here, we present a web server that enables searches for miRNA precursors that can be recognized by diverse RNA-binding proteins based on known sequence motifs to facilitate the identification of other proteins involved in miRNA biogenesis. The database used by the web server contains known human, murine, and *Arabidopsis thaliana* pre-miRNAs. The web server can also be used to predict new RNA-binding protein motifs based on a list of user-provided sequences. We show examples of miRNAmotif applications, presenting precursors that contain motifs recognized by Lin28, MCPIP1, and DGCR8 and predicting motifs within pre-miRNA precursors that are recognized by two DEAD-box helicases—DDX1 and DDX17. miRNAmotif is released as an open-source software under the MIT License. The code is available at GitHub (www.github.com/martynaut/mirnamotif). The webserver is freely available at http://mirnamotif.ibch.poznan.pl.

## 1. Introduction

MicroRNAs (miRNAs) are short, non-coding post-transcriptional gene regulators that control many fundamental cellular processes, such as metabolism, cellular proliferation, apoptosis, immune function, epigenetics, and neurodevelopment [1,2]. The role of microRNA in pathological processes, including human diseases and cancer, is also increasingly acknowledged [3,4,5,6]. Many computational methods are utilized to better understand miRNAs function [7,8,9,10,11,12,13]. 

Most mammalian miRNAs are produced in the canonical miRNA biogenesis pathway. This pathway includes the excision of mature miRNA from the primary transcript (pri-miRNA) by sequential nuclear (Microprocessor complex-mediated) and cytoplasmic (Dicer-mediated) processing through the intermediate miRNA precursor (pre-miRNA) [14,15,16]. Increasing numbers of other cellular proteins and RNAs (including long non-coding RNAs) are shown to influence miRNA biogenesis and function [17,18,19,20], adding to the complexity of the miRNA pathway. Interestingly, some of the RNA-binding proteins (RBPs) known to bind mRNAs can also interact with miRNA precursors, creating indirect crosstalk between mRNA and miRNA [21]. 

**Figure 1:**
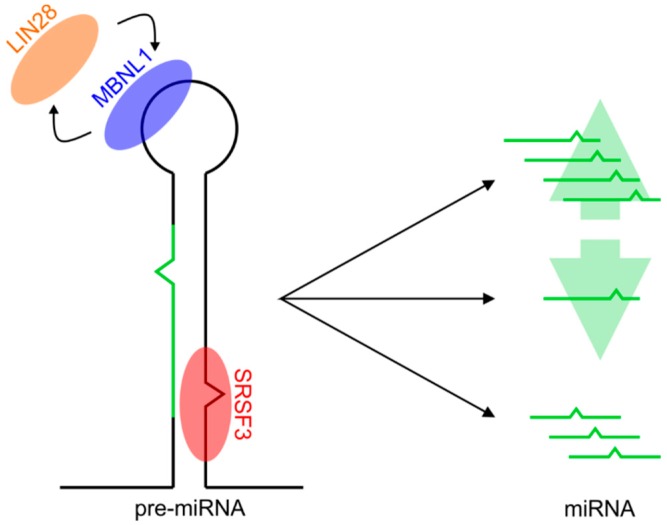
RNA-binding proteins (RBPs) may interact with microRNA (miRNA) precursors through sequence motifs found within the terminal loop (like competing LIN28 and MBNL1) or other elements (like SRSF3). RBPs binding to pre-miRNA precursors may positively or negatively regulate miRNA biogenesis resulting in increased or decreased mature miRNA levels, respectively. Alternatively, protein binding may influence the secondary structure of the miRNA precursor, leading to changed specificity of the Drosha or Dicer cleavage.

The characteristic sequence motifs in the terminal loop or stem portion of pre-miRNA can be selectively recognized by RBPs, which can regulate miRNA production and play an important role in RNA metabolism (Figure 1) [22]. Known protein regulators of miRNA biogenesis were recently reviewed by Michlewski et al. [23]. The protein domains that recognize characteristic sequences in the pre-miRNA terminal loop bind mainly to short single-stranded RNA (ssRNA) regions. These proteins perform various functions in miRNA biogenesis, including miRNA precursor relocation, recruitment, or displacement of other proteins with catalytic activity, or the induction of changes in RNA secondary structures [24]. Different proteins may compete to bind to the same terminal loop [25]. Known sequence motifs found in the terminal loop or stem portion of miRNA precursors are shown in Table 1.

Predicted motifs within miRNA precursors may be validated using RNAs containing mutations within potential protein binding sequences [34]; however, the current method of choice is high-throughput crosslinking and immunoprecipitation followed by sequencing (CLIP-seq) to find the exact sequences interacting with candidate proteins [39,40]. For example, DDX17 was demonstrated by CLIP-seq to bind to multiple miRNA precursors [35].

Adding to the complexity of miRNA biogenesis, multiple SNPs and mutations were identified within miRNA precursors influencing their processing and/or function [41,42]. Small sequence changes may influence the binding of proteins due to recognition motif disruption/creation or structure rearrangement [43,44]. Small sequence changes in microRNA precursors may also influence Drosha and Dicer cleavage sites and the homogeneity of produced mature miRNA [45,46].

miRNAmotif is the first application that enables the user (i) to search for specific motifs in the terminal loop, mature miRNA, linking sequence (sequence between mature miRNAs from two arms), or in whole pre-miRNA among sequences in a widely used miRNA database (miRBase) and (ii) to predict enriched motifs within miRNA precursors’ sequences provided by the user. miRNAmotif is a simple and user-friendly application that gives researchers the opportunity to analyze known pre-miRNAs sequences for the presence of specific motifs that can be recognized by protein regulators of miRNA biogenesis.

## 2. Results

To show the applicability of the miRNAmotif tool for specific biological problems, we analyzed publicly available data to identify pre-miRNAs that may be regulated by Lin28 (Section 2.1.1), MCPIP1, and DGCR8 (Section 2.1.2), and to find the motifs that are potentially recognized by the DDX1 and DDX17 proteins (Section 2.2.1).

### 2.1. Searches for Known Motifs within Pre-miRNA Sequences

#### 2.1.1. Human miRNAs with the Lin28 Motif

We used miRNAmotif to identify pre-miRNAs that contain the GGAG motif within their loop or linking sequence recognized by Lin28 in human miRNAs.

First, we searched for the GGAG motif in the 5′→3′ direction only in the terminal loop. We found 70 pre-miRNAs potentially bound by the Lin28 protein (Supplementary Table S1). Next, we searched for the GGAG motif in the linking sequence in the same direction. We found 155 pre-miRNAs that have the GGAG motif within the sequence between mature miRNAs (Supplementary Table S2). As Lin28 is known to regulate the let-7 family of miRNAs and miRNA-9 [47,48], we investigated whether the miRNAmotif algorithm found the motif in the corresponding precursors. In the first search (loop only), we found hsa-let-7a-2 and hsa-mir-9-1. In the linking sequence search, we showed that in addition to these two miRNAs, the GGAG motif was found in other miRNAs from the let-7 family: hsa-let-7a-1, hsa-let-7c, hsa-let-7d, hsa-let-7e, hsa-let-7f-1, hsa-let-7f-2, hsa-let-7g, and hsa-let-7i.

Next, we analyzed the GGAG motif occurrence in whole pre-miRNA sequences of human pre-miRNAs. As expected, we obtained a long list (793) of pre-miRNAs containing the simple four-letter motif in the 5’→3’ orientation (results not shown). Pre-miRNAs found in the loop-based and the linking-sequence-based search were included in the long list obtained in the whole precursor-based search. However, as Lin28 binds to single-strand regions, most of these pre-miRNAs (with the GGAG motif within a stem) are probably false positive.

Lin28 proteins were previously analyzed in the context of pre-miRNA interactions in multiple model organisms, as these highly conserved proteins are involved in the regulation of pluripotency in stem cells. It was shown in *Xenopus* embryos that Lin28 can also regulate other miRNAs in addition to let-7 family, such as mir-17∼92 and mir-106∼363 cluster miRNAs [49]. One of the regulated miRNAs was xla-mir-363. Analogously, we found hsa-mir-363 to be potentially interacting with Lin28 using miRNAmotif. Another pre-miRNA precursor found in *Xenopus* research and miRNAmotif results is mir-200b, also expressed in miRNA cluster. These results show that other detected pre-miRNAs, besides the known let-7 family and miRNA-9, may interact with Lin28.

Lin28 proteins are found mainly in testis and placenta tissues [50]. Using miRmine, an miRNA tissue-specific expression database [51], we searched for miRNAs that had expression in testis to further limit the list of found pre-miRNAs that can interact with Lin28. As expected, all let-7 family miRNAs had detectable expression in testis according to miRmine. We found that 40 (Table 2) out of 151 pre-miRNAs having the GGAG motif within a linking sequence had expression in testis. Four of the analyzed pre-miRNAs were not included in the miRmine database. In placenta, we found 36 pre-miRNAs showing detectable expression and the set of two groups (testis and placenta) contained 43 unique pre-miRNAs.

#### 2.1.2. Human miRNAs Containing Highly Similar Motifs

SNPs and mutations are widely known to influence miRNAs processing and function. To show that miRNAmotif can be a useful tool for the precise analysis of motifs differing in one nucleotide, we used motifs recognized by MCPIP1 and DGCR8 proteins (UGC and UGU, respectively) as examples.

First, we searched for both UGC and UGU motifs together with an overlap option in the terminal loop only. We found 244 pre-miRNAs with the UGC motif, which may be recognized by the MCPIP1 protein (Supplementary Table S3a), and 315 pre-miRNAs with the UGU motif, which may be recognized by DGCR8 (Supplementary Table S3b). We also found 47 pre-miRNAs containing both UGC and UGU motifs in their terminal loops (Supplementary Table S3c). Then, using the module to search a single motif, we found 606 pre-miRNAs containing the UGY motif in their terminal loops. This number was equal to that of the first search and, more importantly, miRNAs found by searching for the UGY motif were exactly the same as those found previously using two motifs. This result shows that miRNAmotif is a useful tool for the precise analysis of short motifs with a single-nucleotide difference as well.

### 2.2. Predicting Protein-Binding Motifs within Pre-miRNA Sequences

#### 2.2.1. Common Motifs in Pre-miRNAs Interacting with DEAD-Box Helicases

Next, we focused on the search of new motifs potentially interacting with DEAD-box helicases. To perform the first analysis, we used the data generated for pre-miRNAs interactions with one of the DEAD-box helicases, DDX1 [34]. A total of 25 pre-miRNA sequences were analyzed (Table 3).

We searched for the motif in the whole pre-miRNA sequence in the 5’→3’ direction. It was shown that DDX proteins (including DDX1) can interact with sequences outside the terminal loop of the miRNA precursors. As a set of negative sequences, we used all human pre-miRNA sequences. Our software detected five significantly enriched motifs—CUAAYACU (*p* = 1.7 × 10^−7^), CGYUUU (*p* = 5.5 × 10^−7^), CUGKUAA (*p* = 1.4 × 10^−6^), CRUCUUAC (*p* = 1.4 × 10^−6^), and AAUCRU (*p* = 1.1 × 10^−5^) (Supplementary Table S4). Three out of five found motifs harbor the dinucleotide AA detected by Han et al. [34]; however, the miRNAmotif results showed that this short two-nucleotide motif might not be adequate or sufficient for protein binding. The miRNAmotif tool also detected a single significantly enriched motif within the terminal loop in the 5’→3’ direction with all human loops used as negative sequences (UUUAU, *p* = 9.9 × 10^−5^). 

In the next step, we analyzed which miRNAs outside of the experimental group of miRNA precursors could be recognized by DDX1 based on the most significant predicted motif, CUAAYACU. We found seven human miRNA precursors, of which five were included in the experimental group (Supplementary Table S5).

To validate the miRNAmotif function for longer known motifs, we focused on DDX17. We used the data generated for DDX17 interaction with pre-miRNAs. A total of 153 pre-miRNAs sequences were analyzed [35]. Seven pre-miRNAs from the original list (hsa-miR-3648/3687/6087/7641-1/7641-2/3792/3653) had to be excluded from the analysis, as they are no longer included in the new version of the miRBase database or may relate to multiple records in miRBase.

First, miRNAmotif was used to search for motifs located within the terminal loop. The software did not find any significant motifs enriched in the terminal loops of provided sequences in reference to all human pre-miRNA terminal loops. Similarly, no significantly enriched motifs were found in linking sequences. A second search was performed using whole pre-miRNA sequences. Using these settings, the miRNAmotif algorithm detected two significantly enriched motifs, SSGGG (*p* = 6.8 × 10^−9^) and CCSS (*p* = 4.7 × 10^−7^). Next, we analyzed which precursors outside of the experimental group of pre-miRNAs contain the most significantly enriched SSGGG motif. We found 26 pre-miRNAs (with three included in the experimental group) with the SSGGG motif in their terminal loops and 410 pre-miRNAs containing the searched motif in the whole precursor sequence. It is worth mentioning that Moy et al. did not find any enriched motif for DDX17 using CLIP-seq data in their study; however, they restricted their analysis to hexamers only [35]. This proves that miRNAmotif may expand previously performed analyses.

## 3. Discussion

The identification of RNA–protein interactions is a key step towards improving our understanding of the post-transcriptional regulation of gene expression. The number of known motifs recognized by various RNA-binding proteins is increasing due to the rapid development and application of high-throughput sequencing techniques. These motifs are usually considered in the context of mRNA sequences, but can be also ubiquitous in non-coding RNAs, including long non-coding RNAs and miRNA precursors. 

Protein binding, specifically to a group of pre-miRNAs, may influence the efficiency of miRNA biogenesis and explain the differences in cellular levels of various miRNAs. Furthermore, specific protein binding can help improve our understanding of tissue- and cell-specific miRNA-mediated mRNA regulation, as was recently highlighted in two protein–miRNA interaction studies [33,52]. With increased knowledge of miRNA and RBP tissue-specific expression levels, in both physiological and pathological states [53], we can better evaluate which interactions may actually happen and in what cell types, as we showed for Lin28 interactions. In addition to cell- and tissue-specificity, in predicting pre-miRNA–protein interactions, the subcellular localization of protein and pre-miRNA should be also taken into account. In particular, non-ubiquitously expressed proteins that are localized in specific cellular compartments where pre-miRNAs are not present would not interact with these RNA molecules even if a recognized motif was found in the miRNA precursor.

We acknowledge that our software has several limitations. In its current form, it does not take into account the subcellular localization of RBPs and tissue-specific expression levels of specific miRNAs. Future improvements of the algorithm may therefore include a tissue-specific version of the tool, as was previously shown in splicing research [54]. Moreover, like all in silico analyses, miRNAmotif results need to be verified experimentally. This applies especially to short (e.g., two-nucleotide) motifs that may be found in the long list of pre-miRNA precursors. The miRNAmotif tool aims to facilitate and ease the search for miRNA precursors with specific sequence motifs for further evaluation.

Taken together, miRNAmotif is a useful and easy-to-employ application that may increase our understanding of miRNA biogenesis by searching for protein-interacting motifs in pre-miRNA sequences or predicting new motifs in a user-provided group of pre-miRNAs. The miRNAmotif software will be updated with the new releases of the miRBase database.

## 4. Materials and Methods

**Figure 2:**
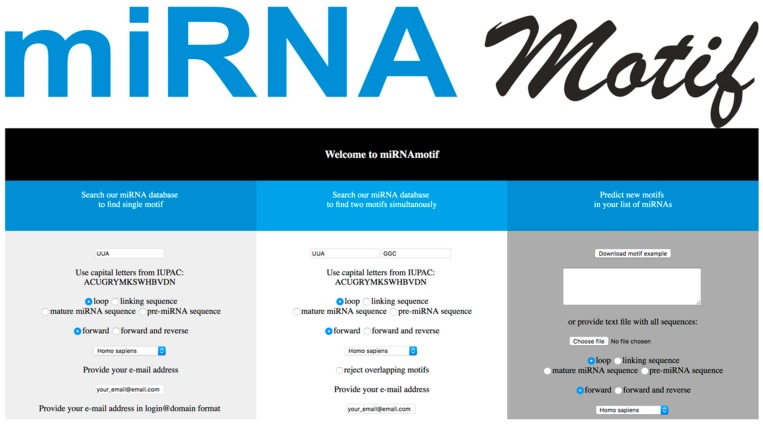
Web server interface enabling three modules available to the user.

miRNAmotif is released as an open-source software under the MIT License. The code is available at GitHub (www.github.com/martynaut/mirnamotif). The web server is freely available at http://mirnamotif.ibch.poznan.pl. miRNAmotif was written in Python programming language using the Pycherry web framework. The software can be used through a user-friendly web interface (Figure 2) or using a command line interface (CLI).

miRNAmotif used sequences of pre-miRNAs extracted from miRBase database version 22 [55]. miRNAs found in humans (hsa), mice (mmu), and *Arabidopsis thaliana* (ath) were included. The mfold algorithm for structure prediction was used to predict secondary structures of miRNA precursors and define the terminal loop portion with a 2-nt overhang [56]; the same algorithm was used to predict the structures presented in miRBase. Linking sequences (sequences between mature miRNAs from two arms) were extracted based on the genomic coordinates of mature miRNAs and their precursors from miRbase. The mfold algorithm was used to predict secondary structures of miRNA precursors and define the passenger strand for pre-miRNAs with defined mature miRNA from a single arm only.

For motif definition, we used IUPAC (International Union of Pure and Applied Chemistry) nucleic acid nomenclature, where R = G or A, Y = U or C, M = A or C, K = G or U, S = G or C, W = A or U, H = A or C or U, B = G or U or C, V = G or C or A, D = G or U or A, and N = G or U or A or C.

Three independent modules are available to the user. The first module is used to search for sequences containing one user-provided motif. The user may choose the organism database of pre-miRNAs, whether the motif should be located within loop, linking sequence, mature miRNA, or anywhere in the pre-miRNA (default: loop), and whether the motif should be searched in one direction (forward, 5′→3′) or in both the 5′→3′ and reverse directions (default: 5′→3′). The .txt and .rft files with sequences that contain the requested motif (marked in red in the .rtf file) are then sent to the user at the indicated e-mail address and printed out on the webpage.

The second module works analogously to the first, but the user can search for two motifs simultaneously. In addition to the options indicated earlier, the user may exclude miRNAs in which two motifs overlap (default: enable overlapping). As a result, the .txt and .rtf files with pre-miRNA, mature miRNA, and linking or loop sequences that contain a single or both motifs (marked in red in the .rtf file) are sent to the user and printed out on the webpage.

The third module enables the identification of new motifs common to a group of miRNAs that are shown to interact with the chosen protein or that exhibit similar processing. The user needs to paste sequences or the names of miRNAs (in miRBase nomenclature, e.g., hsa-mir-21) into the window or upload a text file with the sequences or names of miRNAs. An exemplary input file is provided for download on the webpage. The motif finder has a similar option as the two above modules, in which the user may find motifs in the 5’→3’ direction or in both the 5’→3’ direction and the reverse direction (default: 5’→3’) and can choose whether the motif needs to be found in the terminal loop, linking sequence, mature miRNA, or in a whole pre-miRNA sequence (default: loop). As a result, the user receives a .txt file with motifs and .eps files with motif representations in Weblogo [57]. For motif prediction, we used the DREME algorithm from the MEME suite [58].

## Figures and Tables

**Table 1:** Known motifs in miRNA precursors recognized by protein regulators.

	Protein	Motif	Protein Domain Type	Reference
**Terminal Loop**	hnRNPA1	UAGGGAW	RRM	[26]
	HuR	AUUUUUAUUUU	RRM	[27]
	KSRP	GGGU	KH	[28]
	Lin28	GGAG	CSD&CCHC-ZnF	[29]
	MBNL1	YGCY	CCCH-ZnF	[30]
	MCPIP1	UGC	CCCH-ZnF	[31]
	DGCR8	UGU	Rhed	[32]
	MATR3	AUCUU	nd	[33]
	ZC3H7	SMTANY	nd	
	YBX1	CAUC	nd	
	TRIM71	UAUAA	nd	
	PTBP1/3	UUUUUCCNUCUUU	nd	
**Stem**	DDX1	AA	nd	[34]
	DDX17	VCAUCH	DEAD box	[35]
	Rbfox	GCAUG	RRM	[36]
	SMAD	CAGAC	MH1	[37]
	CELF1/2	UGUNNNNNNNUGU	nd	[33]
	ZC3H10	GCAGCGC	nd	
	SRSF3	CNNC	RS/RRM	[38]

nd—no data.

**Table 2:** Forty pre-miRNAs having the GGAG motif within a linking sequence that are expressed in testis.

pre-miRNA	
hsa-let-7a-1	hsa-miR-204
hsa-let-7a-2	hsa-miR-30e
hsa-let-7c	hsa-miR-320a
hsa-let-7d	hsa-miR-324
hsa-let-7e	hsa-miR-363
hsa-let-7f-1	hsa-miR-3655
hsa-let-7f-2	hsa-miR-378i
hsa-let-7g	hsa-miR-4286
hsa-let-7i	hsa-miR-4784
hsa-miR-107	hsa-miR-5006
hsa-miR-1236	hsa-miR-5579
hsa-miR-132	hsa-miR-5690
hsa-miR-1323	hsa-miR-629
hsa-miR-139	hsa-miR-6775
hsa-miR-142	hsa-miR-6777
hsa-miR-143	hsa-miR-6834
hsa-miR-149	hsa-miR-7154
hsa-miR-152	hsa-miR-7161
hsa-miR-200b	hsa-miR-7847
hsa-miR-200c	hsa-miR-7975

**Table 3:** Twenty-five pre-miRNAs interacting with DDX1 based on a work by Han et al. [34].

pre-miRNA	
hsa-miR-82	hsa-miR-410
hsa-miR-96	hsa-miR-429
hsa-miR-101	hsa-miR-449a
hsa-miR-129	hsa-miR-487b
hsa-miR-138	hsa-miR-490
hsa-miR-141	hsa-miR-495
hsa-miR-146b	hsa-miR-499
hsa-miR-155	hsa-miR-518e
hsa-miR-200a	hsa-miR-524
hsa-miR-200b	hsa-miR-539
hsa-miR-200c	hsa-miR-542
hsa-miR-376	hsa-miR-590
hsa-miR-376a	

## Supplementary Materials

Supplementary materials can be found at http://www.mdpi.com/1422-0067/19/12/4075/s1.

## Abbreviations

miRNA	microRNA
DDX	DEAD box proteins
RS	Arginine/serine-rich domain
RRM	RNA recognition motif
RBP	RNA-binding protein
IUPAC	International Union of Pure and Applied Chemistry
CLIP	Crosslinking immunoprecipitation
CLI	Command line interface
