# Supplementary material for: miRNAmotif—A Tool for the Prediction of Pre-miRNA–Protein Interactions
Source: Int J Mol Sci. 2018 Dec 17;19(12):4075. doi: 10.3390/ijms19124075 (PMC6321451; doi:10.3390/ijms19124075)
Supplement: Supplementary file 1 [file ijms-19-04075-s001.pdf]

SUPPLEMENTARY MATERIAL

miRNAmotif—A Tool for the Prediction of Pre-miRNA–Protein Interactions

Martyna O. Urbanek-Trzeciak[†,*], Edyta Jaworska[†] and Wlodzimierz J. Krzyzosiak

Institute of Bioorganic Chemistry, Polish Academy of Sciences, Noskowskiego 12/14, 61-704, Poznan, Poland; e.jaworska@qmul.ac.uk (E.J.); wlodkrzy@ibch.poznan.pl (W.J.K.)

* Correspondence: martyna.urbanek@gmail.com; Tel.: +48-61-852-85-03

† These authors contributed equally to this work.

SUPPLEMENTARY RESULTS

1 Human miRNAs containing Lin28 motif - results

miRNA name	miRNA loop sequence (motif in red)
hsa-let-7a-2	UCAAGGGAGA
hsa-mir-107	GCAUGGAGU
hsa-mir-139	GUGGCUCGGAG
hsa-mir-204	CUGAGAAUAUAUGAAGGAG
hsa-mir-152	CUCUGGAG
hsa-mir-9-1	GUGUGGAG
hsa-mir-320a	CGGAGUCG
hsa-mir-200c	GCGGUUGGGAGU
hsa-mir-363	UUGAUGAGUAUCAUAGGAGAAA
hsa-mir-324	GUAAAGCUGGAGAC
hsa-mir-600	AGGCAGUGGAGUUACU
hsa-mir-611	CAGACGGAGAUG
hsa-mir-638	GCGGAGGGC
hsa-mir-920	GUAGGAGCUAAGAC
hsa-mir-1181	CCGGAGCGGG
hsa-mir-1183	AGGAGUUCACU
hsa-mir-1233-1	CUAUGGGAGGGG
hsa-mir-1236	GCAUGGAGC
hsa-mir-1202	UGGGGGAGGCA
hsa-mir-1246	GGAGUGGACACC
hsa-mir-1470	GCAGGAGA
hsa-mir-1471	AGAGGCGGAGCA
hsa-mir-320d-2	UGGAGUCA
hsa-mir-3123	ACAUGGAGAGU
hsa-mir-3127	AGGAGAAGGGACGCU
hsa-mir-3134	AUGGAGUAAAAU
hsa-mir-3185	GGGAGCCAGGCC
hsa-mir-320e	GGAGUCG
hsa-mir-4296	CUGAAGUGGUUGUGGGGAGGG
hsa-mir-4313	GCUGGAGGAUGAGGAGAUGC
hsa-mir-4323	GGAGACGCC
hsa-mir-4263	GGAGUGGCC
hsa-mir-4286	AAGUUAGGAGAUGUU

hsa-mir-4291	GUGGAGGC
hsa-mir-1233-2	CUAUGGGAGGGG
hsa-mir-3622a	AGGGAGCU
hsa-mir-3692	UGGAGCAAUAGAUACA
hsa-mir-4418	UUGUGGGAG
hsa-mir-4430	GGAGAUCGUACC
hsa-mir-4480	ACCCAGGGAGUAAGU
hsa-mir-4481	GGAAGGGAGACC
hsa-mir-4495	AGUGGAGUUAUUUU
hsa-mir-4530	GUCAAGGAGC
hsa-mir-4659a	UUGGAGAAA
hsa-mir-499b	GUGGAGAGGA
hsa-mir-4757	CUGCGGGAGG
hsa-mir-4793	GGGAGGCCAAGAAGACC
hsa-mir-5191	CUGGGAGG
hsa-mir-5195	GGAGACCUGACC
hsa-mir-5689	CAGGAGGGUG
hsa-mir-5690	AGGAUUUGGGAGUUAUACU
hsa-mir-6512	GCUGAGGGAGAUGC
hsa-mir-6769a	GGAGCCC
hsa-mir-6778	UGGAGGAAGCA
hsa-mir-6786	CUCAGGAG
hsa-mir-6801	GCCAGGAGC
hsa-mir-6807	GGGGAGAUGUGAAGGAAAGAACU
hsa-mir-6825	CUAGGAGG
hsa-mir-6846	GGAGAGCC
hsa-mir-6847	GGAGUGGGCC
hsa-mir-6850	CGGGAGGGGAAGGGACG
hsa-mir-6871	CUGGAGG
hsa-mir-6884	UCAAGAAAGGGAGAUAGAUGGU
hsa-mir-6891	GCAGGAGC
hsa-mir-7162	GGAGCCCCC
hsa-mir-8052	CUCAGGAG
hsa-mir-8058	GGAGAACC
hsa-mir-10226	ACUGGAGU
hsa-mir-10394	CCGGGAGG

hsa-mir-12118	GUAGGAGGGUGAC

Supplementary Table S1. miRNAs predicted to be recognized by Lin28 (through GGAG motif in loop sequence) in one direction (5’ → 3’).

miRNA name	miRNA linking sequence (motif in red)
hsa-let-7a-1	UUAGGGUCACACCCACCACUGGGAGAUAA
hsa-let-7a-2	UAGAAUUACAUCAAGGGAGAUAA
hsa-let-7c	UAGAGUUACACCCUGGGAGUUAA
hsa-let-7d	UUAGGGCAGGGAUUUUGCCCACAAGGAGGUAA
hsa-let-7e	GAGGAGGACACCCAAGGAGAUCA
hsa-let-7f-1	GUGGGGUAGUGAUUUUACCCUGUUCAGGAGAUAA
hsa-let-7f-2	UUAGGGUCAUACCCCAUCUUGGAGAUAA
hsa-let-7g	UGAGGGUCUAUGAUACCACCCGGUACAGGAGAUAA
hsa-let-7i	GGUCGGGUUGUGACAUUGCCCGCUGUGGAGAUAA
hsa-mir-10226	UGGAGU
hsa-mir-10394	CAUCAACAGUGGUCCCCGGGAGGACUCCACACGCAU
hsa-mir-10525	UGGGAGAAAGUACUCCCGGG
hsa-mir-107	UGGCAUGGAGUUCA
hsa-mir-12118	UUCUAGGGGCUGUAGGAGGGUGACAGUCCUGGA
hsa-mir-1233-1	GGGGGAGCUGCAGGGCUAUGGGAGGGGCCCCAGCGUC
hsa-mir-1233-2	GGGGGAGCUGCAGGGCUAUGGGAGGGGCCCCAGCGUC
hsa-mir-1236	UGGACUGGAAGUGGGCAGCAUGGAGCUGACCUUCAUCAUGGCUU GGCCAACAUAAUG
hsa-mir-1282	CUGCUGCAUGAUCUCCGAGUCCCUGGGGGUAGAGAUGAUGGGGC ACUGGGAG
hsa-mir-132	GUGGGAACUGGAGG
hsa-mir-139	GUGGCUCGGAGGC
hsa-mir-142	AACAGCACUGGAGGG
hsa-mir-143	CAGUUGGGAGUC
hsa-mir-1470	GGGCAGGAGACCC
hsa-mir-1471	AGAGGCGGAGCAC
hsa-mir-149	GUGCUUGUCCGAGGAGGG
hsa-mir-152	CGGGCUCUGGAGCAG
hsa-mir-1910	UUGUGCUGUCCUUGGAGG
hsa-mir-199a-1	AGGAGGCUCUCAAUGUGU
hsa-mir-200b	UGGAGUCAGGUCUC
hsa-mir-200c	GUGCGGUUGGGAGUCUC

hsa-mir-204	GAGAAUAUAUGAAGGAG
hsa-mir-30e	CUGUAAGGUGUUCAGAGGAG
hsa-mir-3123	AUUUCCACAUGGAGAGUGGAGCC
hsa-mir-3134	UGGAGUAAAAUA
hsa-mir-3154	ACGUCAAAGGAGG
hsa-mir-3155b	AGGGAGCUGUCCGGCUCC
hsa-mir-3160-2	UGACCAGCUGAGCUGGAGG
hsa-mir-3168	AGCCUGAGUUGGAGGCUCAUCUUCACUUCUUGCUGU
hsa-mir-3180-4	GCCCCGCUCUGCUGCCGACCCUGUGGAGCGGAGGGUGAAGCCU
hsa-mir-3180-5	GCCCCGCCCUGCUGCCGACCCUGUGGAGCGGAGGGUGAAGCCU
hsa-mir-3202-1	GAUUGGAGUCAUUUUCAGAGCA
hsa-mir-320a	CGGAGUCGGG
hsa-mir-320d-2	UGGAGUCAGG
hsa-mir-320e	CUGGAGUCGGGGA
hsa-mir-324	UAAAGCUGGAGA
hsa-mir-3622a	GCAGGGAGCUGAGC
hsa-mir-363	UGAUGAGUAUCAUAGGAGAAA
hsa-mir-3652	UCCGAACCCAGGGGUGGGGGGUGGAGGCGGCUCCUGCGAUCGAA GGGGACUUGAGACUCACCGGCCGCACGCCAUGAGGGCCCUGUGGG UGCUGGG
hsa-mir-3655	GUUGGAGACUCGAUUGUUGGUGACAGCGAAAGAACGAUAAC
hsa-mir-3689d-1	CUGGGAGGUGUGCUAUCGUCUUCCCCGGG
hsa-mir-3689d-2	CUGGGAGGUGUGCUAUCGUCUUCCCUGGG
hsa-mir-378i	UGGAGUUCUGGGUGCUGUUUUCCCACUC
hsa-mir-3976	CAUAUUGGAGUUGGACUGCAGGGCUUCCUUUACACAAUAAAUAU UGUAUGAAGUGCUGAUGUAACCUUUACUGCAGCAUG
hsa-mir-3978	GGAGCCAAAAUUAGAAGGGCCAAAAUUCUACCUGGCCCACUACC
hsa-mir-4257	AGGAGCUAAGAAGCCCCUACAGGGC
hsa-mir-4259	GGCUCUGAGUGGGGAAAGUGGGGGCCUAGGGGAGGUCA
hsa-mir-4263	GAGAUUGGAGUGGCCAGUGUUCCUAAACA
hsa-mir-4286	AUAGUCAUAAGUUAGGAGAUGUUAGAGCUGU
hsa-mir-4291	GGGUGGAGGCAGAG
hsa-mir-4296	GAGGGCUGAAGUGGUUGUGGGGAGGGGCUUCUG
hsa-mir-4311	ACCUGGAGCAGCUCAGGAGGGCUUCCUGGGUGAGGUGGCAGGU
hsa-mir-4312	CCCCAGAGGAGUCA
hsa-mir-4313	UGCAGCCCCAGCUGGAGGAUGAGGAGAUGCUGGGCUUGG
hsa-mir-4323	UGGAGACGCCAGG

hsa-mir-4325	GAGAUGCUUCUAGAUCCAGGAGGCAGACCUCAAGGAUGGAGAGAAGGCAGAUCC
hsa-mir-4330	GCAGGAGAGGAAGGGGGCUUCUUUUUGACGCCUACUUCAUCAGCUGCU
hsa-mir-4418	UGUGGGAGU
hsa-mir-4436b-1	CAAAUGGUGGAGCAGAUUCGAGGGG
hsa-mir-4436b-2	CAAAUGGUGGAGCAGAUUCGAGGGG
hsa-mir-4437	AGGAGGGGAUGACCCUUG
hsa-mir-4441	AGGGAGGAGACUGUACGUGAGAGAUAGUCAGAUCCGCAUGUUAGAGCAGAGUCUCCUUCGUGU
hsa-mir-4453	UCCUUCGGGGCAGGUGGGGACUGCUCCUUUGGGAGGAAGGAGGA
hsa-mir-4480	CCCAGGGAGUAAGU
hsa-mir-4481	UUUUAAGAGGAAGGGAGACCUAAGCUAG
hsa-mir-4489	CUGGGGACUGGAGAAGU
hsa-mir-4495	CAGUGGAGUUAUUUUGAG
hsa-mir-4497	GCCGGCGGCCGGGAGAUCCGCGCUUCCUGAAUCCCGGCCGGCCCGCCCGGC
hsa-mir-4502	UUUGCUGAUGGAGGGUCUUGCCUCCAUGGGGAUG
hsa-mir-4516	AGGGAGGGCAGGGCAGGCUCUGGGGUGGGGGGUCUGUGAGUCAGCCACG
hsa-mir-4530	AAGGAG
hsa-mir-4636	CCCAGCAAUGGGAGAGUGCU
hsa-mir-4646	CAUUGCGGAGAGGGUCUCAC
hsa-mir-4659a	UUUGGAGAAAAAU
hsa-mir-4667	CAAGAAAAGCUGACUUGGAGG
hsa-mir-4674	GCGCCGCCGGGUCCCUCCUCCCCGGAGAGG
hsa-mir-4687	CUUGGAGCACUUGACCUU
hsa-mir-4690	CCCGUGGGUGAGGAGU
hsa-mir-4708	GAACAGGAGG
hsa-mir-4716	GUAUACAUGUAUACAGGAGG
hsa-mir-4721	AAGCCCCACCAGAAGGUGGAGGCCCAGG
hsa-mir-4725	CACCAGGGAGCUUUCCAUGGGCUG
hsa-mir-4757	CUGCGGGAGGAGAC
hsa-mir-4776-2	CUACUGUCCUUUCAGGAGAGCC
hsa-mir-4784	GAGUGUCAUGGUGGAGCCUC
hsa-mir-499b	CGUGGAGAGGAGUUA
hsa-mir-5006	GGGAGCCCCAUUUACAGUGGUAACUUCC
hsa-mir-5093	UGAGCUAGGAGGAUU
hsa-mir-5191	CCUGGUCUGGGAGGAUAGAAGAG

hsa-mir-557	UGCUGGAGAAGU
hsa-mir-5680	CCUGGACACACAGGAG
hsa-mir-5689	UACUCAGGAGGGUGAGUA
hsa-mir-5690	AUUUGGGAGUUAUAC
hsa-mir-593	CCGUUUCCCUCUGGGGGAGCAAGGAGUGGUGCUGGGUU
hsa-mir-600	AGUGGAGUU
hsa-mir-607	AGGGAGCCAGACUGCCUGG
hsa-mir-6076	AGGUAGGCGAGAGUAAUAUAAUUUCUCCAGGAGAACAUCUGAGA GGGGAAGUUGCUUUCCUGCCCUGGCCCUU
hsa-mir-6083	GAAAGGGAGCAGGAGCAUCGUCUUUAAGAGGGUCAGGUACCUUU GCU
hsa-mir-611	ACGGAGAU
hsa-mir-623	UGCAUCCUAAGCUGUGCUGGAGCU
hsa-mir-629	UUUACGGUGAACCAGGAG
hsa-mir-638	AGGGCGCGGAGGGCGGACCG
hsa-mir-646	CACCUGUGAUCCCAGGAGAGG
hsa-mir-6505	AUGUUGAGCCCUGUCUCUGGGGAGC
hsa-mir-6512	AGGCUGAGGGAGAUGCCUUC
hsa-mir-6740	AACUUGAGGUCUCUGGGAGUUGCUUAAACCAGUUGACCGUAACC UGGCCAGAGAAUUCUGAUAG
hsa-mir-6745	ACUGAUUGCCCCUAGUGGCUAGCCCUGGGUCUAGCAGCCUAUGGC AGUGUCUGGUAAACACUGGUGGAGUCC
hsa-mir-6753	UCAUCUCACGUCAGAGAGAGGGGAAGGGGCUGCCCAGUGAGCCCC CACAGGGCUCUACAUCUCCAGCUGGGCCUGGCUGGAGAUCCCAGG GUCCCUGAAGGCCCCCGCCACCGUUC
hsa-mir-6775	ACAGGAGAGGGCUCACCC
hsa-mir-6777	GAUGGAGAGCCCUGAGCC
hsa-mir-6778	GUGGUUGGAGGAAGCAGCCUGAACC
hsa-mir-6784	ACCCCCGGAGUCUGUCACGG
hsa-mir-6785	GGAGAGGAGCCCCACUGUGGAAGUCUGACCCCC
hsa-mir-6789	CGGGUCCCAGGCUGGGCCCCUCGGAGGCCGGGUGCUCACUGCCCC GUCC
hsa-mir-6792	UGUGAGGAGCAACAGGCACC
hsa-mir-6801	GAGUUAGCCAGGAGCUUUGCAUACUC
hsa-mir-6807	CUGUGGGCAGGGGGAGAUGUGAAGGAAAGAACUAGGACCCAUUC AUC
hsa-mir-6825	GGGGCUAGGAGGCCCC
hsa-mir-6834	GGCGAGGAGGGACCUGUACUAGCCAUGGUUCUGAUCACA
hsa-mir-6843	GAGGGCAGUGAGGCCCUGGGGAGCUGCUGCAGGCAGCAGGUGGG CGGGACGCCAGCAGGCUGUCUAGCUGUUCCCAUG
hsa-mir-6846	AGGAGAGCCCAC

hsa-mir-6847	UGGAAGGAGUGGGCCU
hsa-mir-6850	GGAGGGGAAGGGACG
hsa-mir-6860	GGAGUCGGUGGGUGGAGCCAA
hsa-mir-6869	UCGGAGUACCUCUGC
hsa-mir-6871	UGGAGGU
hsa-mir-6876	GGAGGUGGCACUGCUGUGUGUG
hsa-mir-6884	GCUCAAGAAAGGGAGAUAGAUGGUAG
hsa-mir-6891	UCAUAUCUCUUCUCAGGGAAAGCAGGAGCCCUUCAGCAGGGUCAG GGC
hsa-mir-6892	AAAGCAGGGCUCAGGGCCAGAGAGACUGGGCAUAGAACUAAGGA GGAUGGUGUCCUCCUGACUGCAUCUCUCU
hsa-mir-7154	AGCCUUGGUAGGGAAGCAAGCUGAGGAGAU
hsa-mir-7161	GUUCUCACGCAAAGUGGCCAGGGUGUGGGAGAC
hsa-mir-7162	AACCCCUGCCCCUGGAGCCCCCAGCAGGGCCC
hsa-mir-7847	CACUGCUCAGUGGAG
hsa-mir-7975	ACAGGGGAUUUAUCUCCCAAGGGAGGUCCCCUG
hsa-mir-8052	UCAGGAG
hsa-mir-8055	AAAGAUGGAGUCGUUUGACGUCUUUUAACACC
hsa-mir-8058	CCCACGGAGAACCAGGGCAC
hsa-mir-8080	GAGCCCUUUGAUAAGGGGGAGG
hsa-mir-8082	GGGAGAGAGUCCUCUUUU
hsa-mir-8086	UAGCUUUUUUUUUUUUUUGAGAUGGAGUCUGGC
hsa-mir-9-1	GUGGUGUGGAGUCUUC
hsa-mir-920	GAUGUGUAGGAGCUAAGACACACUCCA
hsa-mir-922	UGGACUGGGGUCAGACUGUGCCCCGAGGAGAA
hsa-mir-936	AAAUCACUCCAGGAGCAACU
hsa-mir-9500	AUAGGAGGGACAGCGGCCUUUCCAACAGG

Supplementary Table S2. miRNAs predicted to be recognized by Lin28 (through GGAG motif in linking sequence) in one direction (5' → 3').

2 Human miRNAs containing MCPIP1 or DGCR8 motif - results

miRNA name	miRNA loop sequence (motif in red)
hsa-let-7a-3	GCUCUGC
hsa-mir-25	GGACGCUGCCCU
hsa-mir-95	GAAAUGCGUU
hsa-mir-105-1	UGGCUGCUCAUGCA
hsa-mir-105-2	UGGCUGCUUAUGCA

hsa-mir-192	AGUGCUCUCGUCUCCCCUCU
hsa-mir-208a	GAUGCUC
hsa-mir-187	GCUGCUCUGA
hsa-mir-199a-2	GGACAAUGCC
hsa-mir-210	UGCCCCA
hsa-mir-135a-1	GAUUCUACUGCUCACUC
hsa-mir-140	GUCAUGC
hsa-mir-144	UUGCGAUGAG
hsa-mir-145	CUUAGAUGCUAAGAUGG
hsa-mir-136	GAUUCUUAUGCUC
hsa-mir-1-1	GGACCUGCU
hsa-mir-155	UGCCUCCA
hsa-mir-219a-2	UCUGCGG
hsa-mir-330	UCUGCAAGAUCAACCGA
hsa-mir-337	UGCACAGUUA
hsa-mir-326	UGGUGCUCA
hsa-mir-339	GUGCCUGC
hsa-mir-422a	UCUCUGCUGC
hsa-mir-450a-1	UGCACUAUAAAUA
hsa-mir-486-1	AGGCCCUUCAUGCUGCCCAGCU
hsa-mir-492	UGCCACCAUUGAGAACG
hsa-mir-493	UUCAUUCGUUUGCACAUUCGG
hsa-mir-512-1	UCUGGUGCCAGAAUGA
hsa-mir-512-2	UCUGGUGCCAGAAUGA
hsa-mir-501	GCUUUCUGAAUGC
hsa-mir-502	GCUGGCUCAAUGC
hsa-mir-505	UUUCUGCCAGUUUAG
hsa-mir-551a	GGGGACUGCC
hsa-mir-552	AGAUGCCU
hsa-mir-551b	UGGUGCAGAACG
hsa-mir-584	UUGCUGGG
hsa-mir-589	GGGUACUGCC
hsa-mir-596	GAACCUGCCUC
hsa-mir-614	AGCCCUGCACU
hsa-mir-615	GGUGCUUAUUG
hsa-mir-618	ACGUACAUGCAGU

hsa-mir-634	UGCCCCA
hsa-mir-651	GUUCAAAUAAAAAUGC
hsa-mir-661	GUUUCGGGCUGC
hsa-mir-654	GCUGAGUUCGUGC
hsa-mir-550a-3	CUUUGCUGG
hsa-mir-767	AGCAUGCUU
hsa-mir-1224	UGGUGCCG
hsa-mir-1468	UGCAUUCAACUCA
hsa-mir-762	GCCAUGC
hsa-mir-670	GAAUUUGCCUU
hsa-mir-764	GCUUGGAAAAUGC
hsa-mir-300	GCUUUACUUGC
hsa-mir-708	UGACUUGCACA
hsa-mir-924	UUGCUUAAG
hsa-mir-935	GCCAUCCUCCGUCUGC
hsa-mir-937	GCUUCGUGC
hsa-mir-943	CUGCUGG
hsa-mir-1200	UUGCCAGA
hsa-mir-1302-2	GGAUGCCC
hsa-mir-1302-3	GGAUGCCC
hsa-mir-1302-4	CAUGCAACAUUUG
hsa-mir-1302-7	GGUGCAAUAAUUG
hsa-mir-1302-8	CAUGCAGUAUUUG
hsa-mir-1304	UGAUCCUGCCA
hsa-mir-1243	GUCCAUCUCCUGC
hsa-mir-1247	GUUGCUCUCUAC
hsa-mir-1250	UUUUGCCUUUUCUAA
hsa-mir-1253	GGACUGCU
hsa-mir-1258	CCUGCGAGUCCCUGG
hsa-mir-1279	CUAAUGCCAAG
hsa-mir-1292	UUGCGUUGA
hsa-mir-1255b-2	UUGCGCCUCAAGAA
hsa-mir-1306	GUGCAGAGG
hsa-mir-1538	CUUCCUGCCGG
hsa-mir-103b-1	UUGAUCCAUAUGCAACAAG
hsa-mir-103b-2	UUGACCUGAAUGCUACAAG

hsa-mir-2116	UCCCAUGCUAAGA
hsa-mir-2681	UGCCCAAAGACUCUUCA
hsa-mir-3125	GGUGCCU
hsa-mir-3132	GGUGCGCC
hsa-mir-3136	UGACUGCA
hsa-mir-3138	GGGUGCCCACAAGACU
hsa-mir-548v	GCCAUCAUGC
hsa-mir-3157	UGCCAACA
hsa-mir-1260b	AGGUGCU
hsa-mir-3175	UGGCCGCGUGCG
hsa-mir-3179-1	GUGCCUUACAGC
hsa-mir-3179-2	GUGCCUUACAGC
hsa-mir-3179-3	GUGCCUUACAGC
hsa-mir-3181	CGUUAGUUGCCCG
hsa-mir-3194	UGCCACCCG
hsa-mir-3196	CUCCCCCAGUGCCAG
hsa-mir-3201	GCUAGGAUUGC
hsa-mir-4293	UGCUAAUUCA
hsa-mir-4303	GCUUGCUCUGC
hsa-mir-4313	GCUGGAGGAUGAGGAGAUGC
hsa-mir-4315-1	GCUUUGC
hsa-mir-4277	GCUGCCCCCACUGC
hsa-mir-4282	UGCCACA
hsa-mir-500b	GCUUUCUGAAUGC
hsa-mir-1302-9	GGAUGCCC
hsa-mir-1302-10	GGAUGCCC
hsa-mir-1302-11	GGAUGCCC
hsa-mir-4315-2	GCUUUGC
hsa-mir-3613	GUUGCAUUUUUAG
hsa-mir-3616	UUUGCCCCAUCAG
hsa-mir-3621	GUGCGGCAGC
hsa-mir-3650	CUGACUGCGUGCCAGG
hsa-mir-3667	CUGCUGGCUGAG
hsa-mir-3670-1	CACCUGCUG
hsa-mir-3686	UCUGCACUGG
hsa-mir-3691	CUGCUGAGGG

hsa-mir-3714	UGCUCCAUCA
hsa-mir-3914-1	GUAUGCUUAAC
hsa-mir-3910-2	GUGCCUUGAU
hsa-mir-3942	UGCGAAGAUAACA
hsa-mir-3944	CGCCUGCG
hsa-mir-3945	UAUAAAAUGCAAUUA
hsa-mir-374c	UGCUAGGACA
hsa-mir-1268b	GUGGGUGC
hsa-mir-378f	CUGCUAAACAACAGAACGAG
hsa-mir-4435-1	AGUGCACUU
hsa-mir-4436a	UCUGCUCCACGA
hsa-mir-4435-2	AGUGCACUU
hsa-mir-4438	CCUUGCCUUUCCUUUGG
hsa-mir-4460	UCUUUGCAGUGA
hsa-mir-4473	GGACUGCU
hsa-mir-4485	ACAUGCGU
hsa-mir-4489	UGAUGCAGGACG
hsa-mir-4493	GAUUAUCACAGCCAUGCCUUU
hsa-mir-4496	UGCCUUUA
hsa-mir-4502	GGGUCUUGCC
hsa-mir-4509-1	UUCCCUUUCUCUUGCCCUGA
hsa-mir-4509-2	UUCCCUUUCUCUUGCCCUGA
hsa-mir-4509-3	UUCCCUUUCUCUUGCCCUGA
hsa-mir-4511	ACUCAGUUUGC
hsa-mir-4524a	CACUGCAGAAUUAUUUUG
hsa-mir-4525	GGGCUGCC
hsa-mir-4526	AGGUGACGUGCU
hsa-mir-4533	GGCUGCC
hsa-mir-378i	GUUCUGGGUGC
hsa-mir-4536-1	UAUAUACUGCCCUGCUUUUAUACAUACAUACAUACAUACCUA
hsa-mir-3973	UGCUCCA
hsa-mir-4633	CAAAUGCGUG
hsa-mir-4645	UCAAGUUGCGA
hsa-mir-4647	GGGAUGCAGAGCC
hsa-mir-4648	UGCCACCCA
hsa-mir-4653	GUUAAGGGUUUGC

hsa-mir-1343	CCUCUGCUCUGG
hsa-mir-4731	UGCAGUCAUCCA
hsa-mir-4756	GCCGAUUCUGC
hsa-mir-4757	CUGCGGGAGG
hsa-mir-4783	UGCGCUAACA
hsa-mir-4785	GCUGCCGC
hsa-mir-2467	GGUCCUGCUCCU
hsa-mir-4799	CUGAGAUGCAGG
hsa-mir-5001	CUGCUGG
hsa-mir-5004	GCACUUGC
hsa-mir-5008	GGUGCCAUCUCC
hsa-mir-5009	UAUUUGCAUUAUACUUA
hsa-mir-5010	GCUGCCUCUUGC
hsa-mir-5089	GAAUCUGCACUC
hsa-mir-5090	GCAAAGCCUGCCCGC
hsa-mir-5093	ACAUGCCAGAGU
hsa-mir-5094	CACUGCCUUUUAUG
hsa-mir-5187	GAAUGCGCUU
hsa-mir-5197	UUUUUGCCAG
hsa-mir-3670-2	CACCUGCUG
hsa-mir-4524b	CAAAAUAAUUCUGCAGUG
hsa-mir-548as	UGCUUUUA
hsa-mir-5580	UGCUGAGAAAAUUCA
hsa-mir-548at	CUGCCAAAAGAAAUGG
hsa-mir-5683	UGCACCA
hsa-mir-5695	AGCAUGCAUU
hsa-mir-5698	AUGCAAAGU
hsa-mir-5705	AGGAUUGCU
hsa-mir-5706	GCUUCUACGUCAUUCAGCACUUGC
hsa-mir-5787	GUGCUAGGUCGGC
hsa-mir-6069	GCCCAGUGC
hsa-mir-6072	GCUACUGC
hsa-mir-6085	UCAUGCAGA
hsa-mir-6088	GUCUUGC
hsa-mir-6089-1	UGCGCCA
hsa-mir-6504	UCUGCACCCUGC

hsa-mir-6511a-1	GCAGAGGGUUGCGC
hsa-mir-6512	GCUGAGGGAGAUGC
hsa-mir-6511b-1	GCAGAGGGUUGCGC
hsa-mir-6730	GCUGCAGCUGC
hsa-mir-6731	UGCCAACCA
hsa-mir-6736	GUUUGCUGGCUGC
hsa-mir-6762	CCUGCAUGG
hsa-mir-6766	GUUCCUUCUCCUGC
hsa-mir-6768	CGGCUGCU
hsa-mir-6773	UAUGCUA
hsa-mir-6799	GGUCACUGACUCUGCUUCC
hsa-mir-6802	AGAUGCCU
hsa-mir-6808	GGGGGCCUUGCU
hsa-mir-6809	GCUCCUCCUGC
hsa-mir-6811	GCAGUGC
hsa-mir-6814	GCCACCCAGCCCUGC
hsa-mir-6817	UGACCUGCCCC
hsa-mir-6819	GUGGGGUGC
hsa-mir-6821	GCCCUGC
hsa-mir-6822	GCUCUGC
hsa-mir-6780b	UGCCUCCA
hsa-mir-6844	GCUAUUUGC
hsa-mir-6845	UGCACACCACCA
hsa-mir-6852	AUGCUCUGACUCCCCUGAU
hsa-mir-6865	GAUGCUUC
hsa-mir-6870	ACUUCAUUUCUCAUGC
hsa-mir-6876	GCACUGC
hsa-mir-6877	GUGCUCAU
hsa-mir-6894	GACAUGCUC
hsa-mir-7111	UCUGCUAUUCGUCCACCAACCUGACUUGA
hsa-mir-7113	CCUCUUGCCAUGG
hsa-mir-7114	CCUGCCACUGG
hsa-mir-6511b-2	GCAGAGGGUUGCGC
hsa-mir-6089-2	UGCGCCA
hsa-mir-6511a-2	GCAGAGGGUUGCGC
hsa-mir-6511a-3	GCAGAGGGUUGCGC

hsa-mir-6511a-4	GCAGAGGGUUGCGC
hsa-mir-7151	UUGGAUUCUGCAAA
hsa-mir-7152	UGCCACA
hsa-mir-7159	GGAAGUAUGCCGGGCC
hsa-mir-7702	ACAUGCAGU
hsa-mir-7847	CUGCUCAG
hsa-mir-7853	UGGUGCA
hsa-mir-8057	CUUGCGG
hsa-mir-8066	GCCUCUGC
hsa-mir-8075	GCUUAUACGUGCCCUUGC
hsa-mir-8078	GAUUCCUGCCUU
hsa-mir-8089	GGCUGCCACC
hsa-mir-548bb	UUGCCCAA
hsa-mir-9718	UCUUAAACCAGUUGCUGA
hsa-mir-3179-4	GUGCCUUACAGC
hsa-mir-3670-3	CACCUGCUG
hsa-mir-3670-4	CACCUGCUG
hsa-mir-548bc	AUUACUGCUCAU
hsa-mir-9986	CUAUUACUACAGCUAUGCCAG
hsa-mir-11401	CUGCUAGG
hsa-mir-12113	GUUCAUUGC
hsa-mir-12116	UGCUUGCCUCCA
hsa-mir-12130	GUGCUUAC

Supplementary Table S3a. miRNAs predicted to be recognized by MCPIP1 (through UGC motif in the loop sequence) in one direction (5' → 3').

miRNA name	miRNA loop sequence (motif in red)
hsa-let-7b	AGUGAUGUU
hsa-mir-19b-2	UAUAUGUA
hsa-mir-20a	AGUGUUUAGUUAUCU
hsa-mir-92a-1	UGUUUCUGUA
hsa-mir-96	UGUCUCUCCG
hsa-mir-101-1	CUGUCUAUUCUAAAGG
hsa-mir-29b-1	AUUUAAAUAGUGAUUGU
hsa-mir-29b-2	AUUUUUCCAUCUUUGUAU
hsa-mir-198	GGUUCCUGUGAUUUUUCC

hsa-mir-30c-2	CUGUGGAAAGUAAGAAAG
hsa-mir-30d	CUGUAAGACACAGCUAAG
hsa-mir-7-3	UGAUGUACUA
hsa-mir-10a	UGUAAGGAAUUUUGUGGUCA
hsa-mir-181b-1	GUGUGGAC
hsa-mir-203a	UCUGUAGCGC
hsa-mir-205	UGUCUCAUACCCAACCA
hsa-mir-222	UGUCUUUCGUAAUCA
hsa-mir-223	UCCAUGUGGUAGA
hsa-mir-23b	UUGUGACUUAAGAUUAAA
hsa-mir-30b	UGUAAUACAUGGAUUG
hsa-mir-122	UGUGUCUAAACUAUCA
hsa-mir-124-1	UUUAAAUGUCCAUACAA
hsa-mir-124-2	UUUAAUGUCAUACAA
hsa-mir-124-3	UUUAAUGUCUAUACAA
hsa-mir-130a	GCACCUGU
hsa-mir-133a-2	GACUGUCCAAUG
hsa-mir-141	UCUAAUUGUGA
hsa-mir-9-1	GUGUGGAG
hsa-mir-9-2	UGUAUUGGUCU
hsa-mir-146a	UGUCAGUGUCA
hsa-mir-149	UUGUCCGA
hsa-mir-154	GCUUUAUUUGU
hsa-mir-184	UGUGACUGUA
hsa-mir-190a	UGUUAUUUAAUCCA
hsa-mir-193a	GAGGGUGUCGGAUC
hsa-mir-194-1	GUGUACCAAU
hsa-mir-29c	GAGUCUGUUUUUGUC
hsa-mir-101-2	CUGUAUAUCUGAAAGG
hsa-mir-34b	CUGUGGUGG
hsa-mir-30e	GGUGUUCA
hsa-mir-26a-2	CUGUUUCCAUCUGUGAGG
hsa-mir-365a	UGUUUCCAUUCCA
hsa-mir-302b	CUGUGACUUUAAAAG
hsa-mir-376c	UAUGUUAUUUAUGGUUA
hsa-mir-372	AUGUCCAAG

hsa-mir-373	CCUUUUUGUCUGUACUGG
hsa-mir-377	GCUUUAUUUAUGU
hsa-mir-380	AUCUCUGU
hsa-mir-340	UAUGUCGUUUG
hsa-mir-151a	UGUCUCAUCCCC
hsa-mir-148b	CUGUGGCUCUCUGAAAG
hsa-mir-335	UUGUCAUAAA
hsa-mir-325	UUUGUGA
hsa-mir-384	UGUAUAAUGUUCA
hsa-mir-429	ACCUGGCCCUCUGU
hsa-mir-449a	AUGUGAAU
hsa-mir-452	ACUUUGUAACUAUGU
hsa-mir-412	UGUUUCUA
hsa-mir-495	GCUUUAUAUGUGA
hsa-mir-193b	GUUUAUGUUUUAU
hsa-mir-497	GCACUGUGGC
hsa-mir-515-1	UUGUCUGAAAG
hsa-mir-520f	UGUGGUCA
hsa-mir-515-2	UUGUCUGAAAG
hsa-mir-525	CUCUUAUGUGAAAAAAAAG
hsa-mir-518f	UUGUCUAAAA
hsa-mir-517a	CUGUUGUAUAAAAGAAAAG
hsa-mir-519d	UGUUUUCUCUUA
hsa-mir-521-2	UUGUCUAAAA
hsa-mir-517b	CUGUUGUCUAAGAAAAG
hsa-mir-520g	UUGUUGUCUGAGAAAAA
hsa-mir-516b-2	GUGUUUUGUGAAAG
hsa-mir-526a-2	CUGUUGAAAGAAAAG
hsa-mir-518e	CUGUUGGCUAAAAGAAAAG
hsa-mir-516b-1	UUGUGAAAG
hsa-mir-517c	CUGUUGUCUAAGAAAAG
hsa-mir-520h	UUGUUGUCUGAGAAAAA
hsa-mir-507	GUGUCUAGAAAU
hsa-mir-509-1	AUGUAUAAUUAAAAAU
hsa-mir-510	AUGUAAUUAGGU
hsa-mir-376a-2	GUGUUUGAU

hsa-mir-553	UUUUGUCUGA
hsa-mir-92b	UGUUUUUUCCCCCGCCA
hsa-mir-563	AUGUAAU
hsa-mir-571	AGCCAUGUCU
hsa-mir-573	ACUCAUGU
hsa-mir-581	GCUUUUAGAAAAUUUGU
hsa-mir-582	ACUAAUCUAACUAAUUGU
hsa-mir-586	CAAUACAUGUG
hsa-mir-587	GGGAAUGUGUC
hsa-mir-588	ACUAUUCCAUUGU
hsa-mir-550a-1	UUGUUGUAAGA
hsa-mir-550a-2	UUGUUGUCAGA
hsa-mir-591	UAGUGUAGAAUG
hsa-mir-595	UUGUAAG
hsa-mir-602	GUGUUCAC
hsa-mir-625	UGUAAUUAGAUCUCA
hsa-mir-627	GGUUGUUU
hsa-mir-33b	GUGUGAGGC
hsa-mir-641	UGUCCUCU
hsa-mir-646	UGUGAUCCCA
hsa-mir-649	AUCUAUGU
hsa-mir-548d-1	CCUGUAAAAGUAAUGG
hsa-mir-663a	CCUCGUGUCUGU
hsa-mir-411	GCUUUAUCUGUGA
hsa-mir-549a	UGUCUCAAAUCA
hsa-mir-542	ACCAGUGU
hsa-mir-671	GAUGGAUGUUU
hsa-mir-668	UAAUGUG
hsa-mir-1323	CUGUGGUUUGAAAGG
hsa-mir-454	GUGUAAAU
hsa-mir-1298	UACCCUGUGUUA
hsa-mir-892a	ACUUACACUACAUGU
hsa-mir-509-2	AUGUAUAAUUAAAAAU
hsa-mir-450b	AUGUAAUAUAAGU
hsa-mir-890	GCUUAGAUUACAUGU
hsa-mir-888	ACUUAGAUUACAUGU

hsa-mir-875	UGUUCUUAAAAUCA
hsa-mir-876	UCUAAGCUAAUGU
hsa-mir-543	GCUUUAUUUGUGA
hsa-mir-922	CAGACUGUG
hsa-mir-509-3	AUGUAUAAUUAAAAAU
hsa-mir-940	CCGUGUGUUGAGG
hsa-mir-942	UGUACUCACAGCCCCUCA
hsa-mir-944	UUAAAUUGUAUAAAGA
hsa-mir-1231	UGUCUGUCCA
hsa-mir-663b	UCUGUGG
hsa-mir-1291	UUGUACUGUGG
hsa-mir-1293	AGCUUGUACCU
hsa-mir-1294	CAUUGUCUG
hsa-mir-1295a	GAAAUGUGGCCUG
hsa-mir-1302-5	CAUGUAAUCUUUG
hsa-mir-1305	UUGUAAAGA
hsa-mir-548f-5	UGUAAGUA
hsa-mir-1244-1	ACUUAGCUGU
hsa-mir-1245a	GAUGUUGAAUACUCUUU
hsa-mir-1248	ACCAUGU
hsa-mir-1257	CCCUGUGG
hsa-mir-1265	GGCAUGUUCA
hsa-mir-1268a	CCUGUGG
hsa-mir-1275	UGUGUCUGUA
hsa-mir-1281	GAGUGUCUCUUGUC
hsa-mir-1197	GCUUUAUUUGUGA
hsa-mir-1537	GUCCUGUCCACAC
hsa-mir-1908	AUGUGUGGU
hsa-mir-1972-1	AUGUGUCAUUUAAAAU
hsa-mir-1976	CCUAAGGUGU
hsa-mir-2052	AUGUCCCUUUAGUUCAAAGU
hsa-mir-2053	CUGUGAAG
hsa-mir-2278	GUGUGGAC
hsa-mir-2909	CUUUCCCCUGUGG
hsa-mir-3119-2	AGAUUGUUUAGCU
hsa-mir-3120	GGUGAGCGGAUGUUC

hsa-mir-3129	CUGUUAAUGAAU
hsa-mir-3130-1	CUGUUGUCAAG
hsa-mir-466	UGUAUAUAUGUA
hsa-mir-3148	UGAUGUAGCCA
hsa-mir-3074	GUGUGUAAAAUGAGAAC
hsa-mir-3158-2	UCCAAUACCUGUAAGCAGA
hsa-mir-3162	AACAAUGUUU
hsa-mir-3163	ACCUUCCUUCCUUGU
hsa-mir-3166	GAUUUCUGUAUCAUCCUC
hsa-mir-3187	GGUCACCAUGUUCUCC
hsa-mir-3190	GCCCUUGU
hsa-mir-514b	GUGUAAUUAGAU
hsa-mir-4296	CUGAAGUGGUUGUGGGGAGGG
hsa-mir-4294	CCAUGUCUGUGAGGG
hsa-mir-4307	GUUUGUCUAU
hsa-mir-4317	UUUAUUUUGUAG
hsa-mir-4253	ACUUACUUGU
hsa-mir-4269	GCCUCUUUCUUUGU
hsa-mir-4283-1	GGUGUUUCUGUC
hsa-mir-4286	AAGUUAGGAGAUGUU
hsa-mir-1244-2	ACUUAGCUGU
hsa-mir-1244-3	ACUUAGCUGU
hsa-mir-1972-2	AUGUGUCAUUUAAAAAU
hsa-mir-4283-2	GGUGUUUCUGUC
hsa-mir-3609	ACCUUGU
hsa-mir-3614	UCUGUCUUGG
hsa-mir-3615	GGUCUGUGGUCC
hsa-mir-3617	AUUAGAAACCAUAUGU
hsa-mir-3649	AGUGUUUCU
hsa-mir-3654	GAAUGUUC
hsa-mir-3671	UGUAAAUUA
hsa-mir-3684	UGUUGAGCAUUACUCA
hsa-mir-3688-1	UAUGUAUGUCUGUUA
hsa-mir-3689a	UGUGAUCCUGUG
hsa-mir-3690-1	AGGUGUUUCUACU
hsa-mir-3180-4	ACCCUGU

hsa-mir-3180-5	ACCCUGU
hsa-mir-3908	UAACGUGUA
hsa-mir-3916	GGUGAAUGUGUC
hsa-mir-3919	UAGUUUGUAGUAGUUCGUA
hsa-mir-3925	UGUCACAUCUCCA
hsa-mir-3939	GUACCUGUCACAU
hsa-mir-378d-1	GUUGUUAUUAC
hsa-mir-548ab	ACUUGUAUUUAUUUGU
hsa-mir-4418	UUGUGGGAG
hsa-mir-4421	ACACUUGUUGGG
hsa-mir-4422	ACCAUGUACCAGU
hsa-mir-4423	UGUUUAAGCCUA
hsa-mir-4426	UUGUCUGACUACAAUAUUCAAA
hsa-mir-4429	GUGUCUAAU
hsa-mir-4432	UGUCAUCC
hsa-mir-4451	ACCACUUCCACAUGU
hsa-mir-4463	UCUGAAUUUGUGA
hsa-mir-3155b	CUGUCCGG
hsa-mir-4482	GUGUGGAAGAUGGC
hsa-mir-4498	AUCUUUGU
hsa-mir-4501	AAAUAUGUCUGAGCUU
hsa-mir-4505	ACUUUCUGU
hsa-mir-4513	CUGUCUAAAA
hsa-mir-4521	UGUAGCAUCA
hsa-mir-4528	AUAUGUAU
hsa-mir-4637	UGUAUUUA
hsa-mir-4652	ACUAACUAACCAGAACUAUUUUGU
hsa-mir-4654	UGGAAUGUG
hsa-mir-4655	CGUGGGAAAGUGUUG
hsa-mir-4663	GCAUCUGU
hsa-mir-4669	CAUCUGUG
hsa-mir-4679-1	UUGUUAGAAACAAAA
hsa-mir-4681	CAUUGUG
hsa-mir-4684	ACAUUUGUCUUGGU
hsa-mir-4691	GAUGUCUC
hsa-mir-4701	GUGAAUGUCGGGC

hsa-mir-4703	UGUUUGAUUUG
hsa-mir-4704	UGUCUUCCUCACUCA
hsa-mir-4716	UACAUGUA
hsa-mir-4718	ACCUGUUUGU
hsa-mir-4723	AGAGAAUGUUGAACU
hsa-mir-4724	GUGUACAUUAUCUAUUAGAAAAU
hsa-mir-4729	AACUGUGACCUU
hsa-mir-4732	UGUGUGUGUCCA
hsa-mir-4739	UGUCUUCCC
hsa-mir-4768	GGGAACUGUG
hsa-mir-4776-2	UGUCCUUUCA
hsa-mir-4777	ACCUCAUAUGU
hsa-mir-4782	AAAAGAUUUGGUGUU
hsa-mir-4786	UCUCCCUGUGA
hsa-mir-4787	ACGGCCUGU
hsa-mir-4802	AGUGUUCAAGUCU
hsa-mir-5087	GUUAAGUGUUGUCCUAC
hsa-mir-5091	GCGGGUUGUGGGGUUUAGGU
hsa-mir-5188	UGUAAGCAGGAUCCAUUCA
hsa-mir-5190	UUAAUGUUCCACAG
hsa-mir-5581	ACCCUAUACAUACCUGU
hsa-mir-5588	UUUUUUUUUUUUUUUUUUAAUGUUAA
hsa-mir-4536-2	AUGUAUAAAA
hsa-mir-5590	GUGUUGAUCAAC
hsa-mir-5692a-1	CCUCAUGUGU
hsa-mir-5692a-2	CCUCAUGUGU
hsa-mir-5694	CAUAUGUAUCUG
hsa-mir-5696	ACAAUGU
hsa-mir-5701-1	CCUAAGACUUGU
hsa-mir-5702	UGAAAUCCUUCUGUA
hsa-mir-5692b	UGUGUACA
hsa-mir-5707	UGUGAACA
hsa-mir-5701-2	CCUAAGACUUGU
hsa-mir-6068	GCUGAGUGU
hsa-mir-6075	AUGUUCCAGU
hsa-mir-6077	GCUAGUUUGUUUGU

hsa-mir-6080	GGGCUGGUUGUC
hsa-mir-6090	GCGAUGU
hsa-mir-6124	CAAUGUCCUG
hsa-mir-6132	UAUCCGCUGUUG
hsa-mir-6134	UGAUGUCAGUA
hsa-mir-6165	AUCCACCUGU
hsa-mir-6499	CUGUUGGG
hsa-mir-6505	CUGUCUCUGG
hsa-mir-892c	ACUUAGAUUACAUGU
hsa-mir-6732	CCAUCAGUGUGGG
hsa-mir-6733	CCUUCUUGU
hsa-mir-6749	UGUCCUGUCUCACUCA
hsa-mir-6781	GCUUCUCCCUAAUGU
hsa-mir-6784	GAGUCUGUC
hsa-mir-6785	CACUGUG
hsa-mir-6788	AUUGAUUGU
hsa-mir-6806	UCCAUGUGA
hsa-mir-6807	GGGGAGAUGUGAAGGAAAGAACU
hsa-mir-6812	AGUCCUGUCU
hsa-mir-6816	GGGACUGUGACA
hsa-mir-6820	CCUCAUGU
hsa-mir-6823	CUGUGAAAG
hsa-mir-6824	CCUUCACUGU
hsa-mir-6827	CUGUCUCUGAG
hsa-mir-6830	UCCCUCAUCUGUUUCUCACUGG
hsa-mir-6833	AAAAUCCCUGUUAACUU
hsa-mir-6834	CCUGUACUAGCCAUGG
hsa-mir-6835	AUGUUUGU
hsa-mir-6841	GUGUUUUUUAAC
hsa-mir-6859-1	GCGGGUGUCAGC
hsa-mir-6862-1	GAUUUGUCUC
hsa-mir-6864	UGUGGGUCA
hsa-mir-6874	GCUUUAAUGU
hsa-mir-6888	CUGUCUCUGAUUGUUUCCAAG
hsa-mir-6893	CCCACUGUGG
hsa-mir-3690-2	AGGUGUUUCUACU

hsa-mir-7155	GUUGUGACAU
hsa-mir-7852	ACUACUACUUUCAUAUGU
hsa-mir-7855	ACUAUUGU
hsa-mir-7856	CCUUUGUUCUGG
hsa-mir-8073	CGUCAGUGUUUG
hsa-mir-6862-2	GAUUUGUCUC
hsa-mir-6859-2	GCGGGUGUCAGC
hsa-mir-6859-3	GCGGGUGUCAGC
hsa-mir-1244-4	ACUUAGCUGU
hsa-mir-6859-4	GCGGGUGUCAGC
hsa-mir-5701-3	CCUAAGACUUGU
hsa-mir-10395	UGACUGUGACCA
hsa-mir-10527	CUGUUUCCUCUUAUUCAAG
hsa-mir-11399	UGUUUCCCCA
hsa-mir-3085	AUUAUGUGU
hsa-mir-6529	CAGUGUCAAUG
hsa-mir-12129	CCACAAAAUGUUACUAGGG
hsa-mir-12131	ACUGAUGU

Supplementary Table S3b. miRNAs predicted to be recognized by DGCR8 (through UGU motif in the loop sequence) in one direction (5’ → 3’).

**miRNA name**	**miRNA loop sequence (motif in red)**
hsa-mir-17	UGAUAUGUGCA
hsa-mir-19b-1	GCUGUGUGAUAUUCUGC
hsa-mir-23a	UGCUUCCUGUCACA
hsa-mir-26a-1	CUGUGCAGGUCCCAAUGGG
hsa-mir-26b	UGUGCUGUCC
hsa-mir-224	AUUGUGCAU
hsa-let-7i	GUUGUGACAUUGC
hsa-mir-134	AUGCACUGU
hsa-mir-296	UGUGCCUAAUUCA
hsa-mir-135b	UGCUGUCCCA
hsa-mir-346	CUGUUGCUCUGAAG
hsa-mir-500a	GCUGUCUGAAUGC
hsa-mir-487b	CGUUUUGCUCAUGUCG
hsa-mir-572	GCGCCUGUGC

hsa-mir-606	UGUGCCAUCCA
hsa-mir-623	GCUGUGC
hsa-mir-758	GCUUUAUUUGUGC
hsa-mir-1301	CUGUGCUGGGG
hsa-mir-769	GAUGUUGCUCUC
hsa-mir-802	GUGUCCAUCAUGCAAC
hsa-mir-301b	CUGUGCUCUGAGAAG
hsa-mir-1179	UGUAUUGCCU
hsa-mir-1307	UGUGUUGCCA
hsa-mir-1910	UUGUGCUGUCCUUGGA
hsa-mir-3659	UGCUUGUAUA
hsa-mir-3678	GUUUGUUUGC
hsa-mir-4420	UGUGCACA
hsa-mir-4425	GCUUUGCGAAGUGU
hsa-mir-4442	GUGUGUGUGC
hsa-mir-4452	GUAGUGUGCAC
hsa-mir-3689d-1	UGUGCUAUCG
hsa-mir-3689d-2	UGUGCUAUCG
hsa-mir-3689f	UGUGAUGCUGUG
hsa-mir-4499	GUUGUACAUAGAAAUGC
hsa-mir-4539	GCUGUGC
hsa-mir-4749	UGUGCACA
hsa-mir-5000	GUAACUUGUGCUUAUAAC
hsa-mir-5587	GCCCUGUGC
hsa-mir-5591	GCUUAUGUAUGC
hsa-mir-6728	ACUGCCCUGU
hsa-mir-6737	GGUUUGUUUGCUUAUUC
hsa-mir-6746	GAGCUGCUGUC
hsa-mir-6761	GUGUUGC
hsa-mir-6797	CUGUGCUCACUGGG
hsa-mir-6848	GGUGUGCUCUGAUCCCCCU
hsa-mir-7850	CUGUGCAG
hsa-mir-7851	UGUGCCACCA

Supplementary Table S3c. miRNAs predicted to be recognized by both MCPIP1 and DGCR8 proteins (through UGC and UGU, respectively, in the loop sequence) in one direction (5’ → 3’) with overlap option.

3 Common motifs in pre-miRNAs interacting with DDX1

Motif sequence	*p*
CUAAYACU	1.7e-007
CGYUUU	5.5e-007
CUGKUAA	1.4e-006
CRUCUUAC	1.4e-006
AAUCRU	1.1e-005

Supplementary Table S4. Motifs predicted to be recognized by DDX1 within whole pre-miRNA sequence against all human pre-miRNA sequences in one direction (5' → 3').

miRNA name	miRNA precursor sequence (motif in red)	Included in initial experimental group
hsa-mir-141	CGGCCGGCCCUGGGUCCAUCUUCCAGUACAGUG UUGGAUGGUCUAAUUGUGAAGCUCCUAACACUG UCUGGUAAAGAUGGCUCCCGGGUGGGUUC	Yes
hsa-mir-429	CGCCGGCCGAUGGGCGUCUUACCAGACAUGGUU AGACCUGGCCCUCUGUCUAAUACUGUCUGGUAA AACCGUCCAUCCGCUGC	Yes
hsa-mir-548b	CAGACUAUAUAUUUAGGUUGGCGCAAAAGUAA UUGUGGUUUUGGCCUUUAUUUUCAAUGGCAAG AACCUCAGUUGCUUUUGUGCCAACCUAAUACUU	No
hsa-mir-548h-4	GCUAUUAGGUUGGUGCAAAAGUAAUCGCGGUU UUUGUCAUUACUUUAAUUACUUUACGUUUCAU UAAUGACAAAAACCGCAAUUACUUUUGCACCAA CCUAAUACUUGCUA	No
hsa-mir-200b	CCAGCUCGGGCAGCCGUGGCCAUCUUACUGGGC AGCAUUGGAUGGAGUCAGGUCUCUAAUACUGCC UGGUAAUGAUGACGGCGGAGCCCUGCACG	Yes
hsa-mir-200c	CCCUCGUCUUACCCAGCAGUGUUUGGGUGCGGU UGGGAGUCUCUAAUACUGCCGGGUAAUGAUGG AGG	Yes
hsa-mir-200a	CCGGGCCCCUGUGAGCAUCUUACCGGACAGUGC UGGAUUUCCCAGCUUGACUCUAACACUGUCUGG UAACGAUGUUCAAAGGUGACCCGC	Yes

Supplementary Table S5. miRNA precursors predicted to be recognized by DDX1 with most significantly enriched motif (CUAAYACU) in one direction (5' → 3').
